# Supplementary figures and images for: Cohort study of the mortality among patients in New York City with tuberculosis and COVID-19, March 2020 to June 2022
Source: PLOS Glob Public Health. 2023 Apr 26;3(4):e0001758. doi: 10.1371/journal.pgph.0001758 (PMC10132536; doi:10.1371/journal.pgph.0001758)

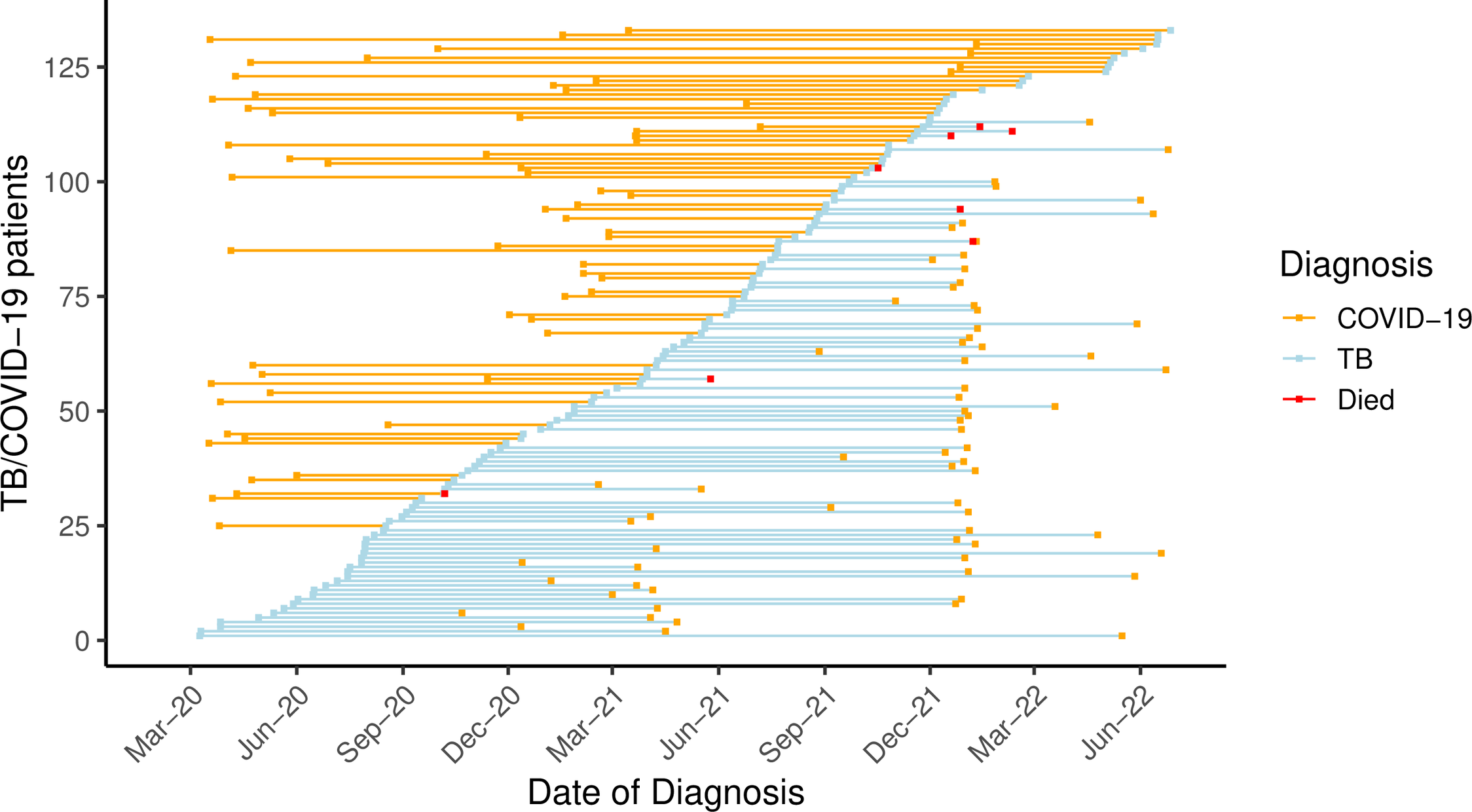

Supplement: S1 Fig — The dates when patients were diagnosed with TB are shown by blue points and the dates when patients were diagnosed with COVID-19 are shown by yellow points. The line between points represents the time between diagnosis of the two diseases. The line is the color of the first diagnosis. Deaths are shown as red points. Nine deaths occurred among these patients; however, one death occurred after 6/30/2022, but before that patient completed treatment for TB, and is thus not visible here. Five deaths occurred during a COVID-19 wave in late 2021. Four of these deaths occurred in a hospital, and COVID-19 was not noted in their death certificates. The fifth person who died was diagnosed with COVID-19 soon after their death. (TIF) [file pgph.0001758.s001.tif]
