# Supplementary material for: Cohort study of the mortality among patients in New York City with tuberculosis and COVID-19, March 2020 to June 2022
Source: PLOS Glob Public Health. 2023 Apr 26;3(4):e0001758. doi: 10.1371/journal.pgph.0001758 (PMC10132536; doi:10.1371/journal.pgph.0001758)
Supplement: S1 File — (DOCX) [file pgph.0001758.s002.docx]

Full results of Cox regression analyses:

Call:

coxph(formula = Surv(time, Died) ~ COVID)

n= 1008, number of events= 128

coef exp(coef) se(coef) z Pr(>|z|)

COVID1 0.7776 2.1763 0.2304 3.375 0.000738 ***

---

Signif. codes: 0 ‘***’ 0.001 ‘**’ 0.01 ‘*’ 0.05 ‘.’ 0.1 ‘ ’ 1

exp(coef) exp(-coef) lower .95 upper .95

COVID1 2.176 0.4595 1.385 3.419

Concordance= 0.545 (se = 0.017 )

Likelihood ratio test= 9.61 on 1 df, p=0.002

Wald test = 11.39 on 1 df, p=7e-04

Score (logrank) test = 11.98 on 1 df, p=5e-04

Call:

coxph(formula = Surv(time, Died) ~ COVID + AGE_AT_EVENT + SEX)

n= 1008, number of events= 128

coef exp(coef) se(coef) z Pr(>|z|)

COVID1 0.962763 2.618921 0.232925 4.133 3.57e-05 ***

AGE_AT_EVENT 0.047165 1.048295 0.005293 8.911 < 2e-16 ***

SEXMale 0.105077 1.110797 0.185259 0.567 0.571

---

Signif. codes: 0 ‘***’ 0.001 ‘**’ 0.01 ‘*’ 0.05 ‘.’ 0.1 ‘ ’ 1

exp(coef) exp(-coef) lower .95 upper .95

COVID1 2.619 0.3818 1.6590 4.134

AGE_AT_EVENT 1.048 0.9539 1.0375 1.059

SEXMale 1.111 0.9003 0.7726 1.597

Concordance= 0.739 (se = 0.023 )

Likelihood ratio test= 104.7 on 3 df, p=<2e-16

Wald test = 88.87 on 3 df, p=<2e-16

Score (logrank) test = 101 on 3 df, p=<2e-16

Testing for correlation of age and diabetes among patients with TB-alone:

Call:

lm(formula = DIABETES ~ AGE_AT_EVENT)

Residuals:

Min 1Q Median 3Q Max

-0.5867 -0.2935 -0.1281 0.4584 0.9546

Coefficients:

Estimate Std. Error t value Pr(>|t|)

(Intercept) -0.1350487 0.0331635 -4.072 4.98e-05 ***

AGE_AT_EVENT 0.0075182 0.0005994 12.543 < 2e-16 ***

---

Signif. codes: 0 ‘***’ 0.001 ‘**’ 0.01 ‘*’ 0.05 ‘.’ 0.1 ‘ ’ 1

Residual standard error: 0.4075 on 1139 degrees of freedom

Multiple R-squared: 0.1214, Adjusted R-squared: 0.1206

F-statistic: 157.3 on 1 and 1139 DF, p-value: < 2.2e-16

Full results of Cox regression analyses for subsets of patients with and without diabetes:

Call:

coxph(formula = Surv(time, Died) ~ COVID + AGE_AT_EVENT + SEX,

data = “Individuals with diabetes”)

n= 248, number of events= 48

coef exp(coef) se(coef) z Pr(>|z|)

COVID1 1.16451 3.20435 0.34276 3.397 0.00068 ***

AGE_AT_EVENT 0.02418 1.02448 0.01197 2.021 0.04332 *

SEXMale 0.13459 1.14407 0.32438 0.415 0.67820

---

Signif. codes: 0 ‘***’ 0.001 ‘**’ 0.01 ‘*’ 0.05 ‘.’ 0.1 ‘ ’ 1

exp(coef) exp(-coef) lower .95 upper .95

COVID1 3.204 0.3121 1.6367 6.273

AGE_AT_EVENT 1.024 0.9761 1.0007 1.049

SEXMale 1.144 0.8741 0.6058 2.161

Concordance= 0.649 (se = 0.043 )

Likelihood ratio test= 12.63 on 3 df, p=0.006

Wald test = 13.88 on 3 df, p=0.003

Score (logrank) test = 14.93 on 3 df, p=0.002

Call:

coxph(formula = Surv(time, Died) ~ COVID + AGE_AT_EVENT + SEX,

data = “Individuals without diabetes)

n= 760, number of events= 80

coef exp(coef) se(coef) z Pr(>|z|)

COVID1 0.750855 2.118810 0.327812 2.291 0.022 *

AGE_AT_EVENT 0.052301 1.053693 0.006198 8.438 <2e-16 ***

SEXMale 0.025514 1.025842 0.229634 0.111 0.912

---

Signif. codes: 0 ‘***’ 0.001 ‘**’ 0.01 ‘*’ 0.05 ‘.’ 0.1 ‘ ’ 1

exp(coef) exp(-coef) lower .95 upper .95

COVID1 2.119 0.4720 1.1144 4.028

AGE_AT_EVENT 1.054 0.9490 1.0410 1.067

SEXMale 1.026 0.9748 0.6541 1.609

Concordance= 0.762 (se = 0.028 )

Likelihood ratio test= 85.29 on 3 df, p=<2e-16

Wald test = 72.8 on 3 df, p=1e-15

Score (logrank) test = 87.98 on 3 df, p=<2e-16
