# Supplementary material for: Cohort study of the mortality among patients in New York City with tuberculosis and COVID-19, March 2020 to June 2022
Source: PLOS Glob Public Health. 2023 Apr 26;3(4):e0001758. doi: 10.1371/journal.pgph.0001758 (PMC10132536; doi:10.1371/journal.pgph.0001758)
Supplement: S1 Table — (DOCX) [file pgph.0001758.s003.docx]

S1 Table. Comparison of mortality among patients diagnosed with TB in NYC between 3/1/2020 and 6/30/2022 (TB-alone group) and patients diagnosed with COVID-19 during the same period (COVID-19 alone group).

| **Characteristic** | | **TB-alone**  **(n=902)** | **COVID-19 alone (n=2,592,760)** | **p-value** |
| --- | --- | --- | --- | --- |
| **Deaths** | | 105 (12%) | 40,848 (2%) | <0.001*** |
| **Deaths, stratified by age** | **0 to 44** | 16/377 (4%) | 1,450/1,661,969 (0.1%) | <0.001*** |
|  | **45 to 64** | 24/253 (9%) | 8,585/638,218 (1%) | <0.001*** |
|  | **65+** | 65/272 (24%) | 30,813/292,573 (11%) | <0.001*** |
