# Supplementary material for: Cohort study of the mortality among patients in New York City with tuberculosis and COVID-19, March 2020 to June 2022
Source: PLOS Glob Public Health. 2023 Apr 26;3(4):e0001758. doi: 10.1371/journal.pgph.0001758 (PMC10132536; doi:10.1371/journal.pgph.0001758)
Supplement: S2 Table — (DOCX) [file pgph.0001758.s004.docx]

S2 Table. Comparison of patients diagnosed with TB in NYC in 2016-2018 (pre-pandemic TB) and diagnosed with TB in NYC between 3/1/2020 and 6/30/2022 (TB-alone group).

| **Characteristic** | | **Pre-pandemic TB**  **(n=1,710)** | **TB-alone**  **(n=902)** | **p-value** |
| --- | --- | --- | --- | --- |
| **Median [IQR] Age in Years at TB diagnosis** | | 49 [32, 65] | 51 [35, 68] | 0.007** |
| **Male sex** | | 1072 (63%) | 583 (65%) | 0.35 |
| **US-born** | | 242 (14%) | 110 (12%) | 0.17 |
| **Race / Ethnicity (among US-born)** | |  |  | 0.70 |
| **Non-Hispanic White** | | 38 (16%) | 15 (14%) |  |
| **Non-Hispanic Black or**  **African American** | | 110 (45%) | 58 (53%) |  |
| **Hispanic** | | 66 (27%) | 25 (23%) |  |
| **Asian** | | 17 (7%) | 9 (8%) |  |
| **Other / Unknown** | | 11 (5%) | 3 (3%) |  |
| **Median [IQR] years living in US+** | | 12 [3, 24] | 12 [4, 25] | 0.64 |
| **Pulmonary involvement** | | 1369 (80%) | 756 (84%) | 0.02* |
| **Cavitary Chest X-ray** | | 314/1369 (23%) | 149/756 (20%) | 0.09 |
| **Ever Sputum Smear** **positive** | | 707/1369 (52%) | 421/756 (56%) | 0.08 |
| **Multi-drug resistant (out of culture positive)** | | 38/1364 (3%) | 12/758 (2%) | 0.10 |
| **History TB disease (documented or self-reported)** | | 120 (7%) | 54 (6%) | 0.36 |
| **Other health problems** | |  |  |  |
| **Diabetes** | | 348 (20%) | 218 (24%) | 0.03* |
| **HIV Status** | |  |  | 0.70 |
| **Infected** | | 84 (5%) | 40 (4%) |  |
| **Uninfected** | | 1367 (80%) | 716 (79%) |  |
| **Unknown/refused** | | 259 (15%) | 146 (16%) |  |
| **Social risk factors within the past 12 months prior to diagnosis** | |  |  |  |
| **Homelessness** | | 59 (3%) | 37 (4%) | 0.44 |
| **Incarceration** | | 20 (1%) | 5 (1%) | 0.14 |
| **Injection drug use** | | 10 (1%) | 2 (0%) | 0.24 |
| **Non-injection drug use** | | 82 (5%) | 61 (7%) | 0.04* |
| **Alcohol abuse** | | 25 (1%) | 24 (3%) | 0.03* |
| **Smoked tobacco** | | 310 (18%) | 148 (16%) | 0.28 |
| **Median [IQR] days from cough onset to TB diagnosis ++** | | 38 [20, 89] | 50 [20, 93] | 0.05* |
| **Ever on Directly Observed Therapy for TB (among** **eligible)** | | 1242/1523 (82%) | 570/761 (75%) | <0.001*** |
| **Hospitalized for TB** | | 877 (51%) | 464 (51%) | 0.97 |
| **Deaths** | | 139 (8%) | 105 (12%) | 0.004** |
| **Deaths prior to treatment initiation** | | 37/139 (27%) | 26/105 (25%) | 0.77 |
| **Deaths, stratified by age** | **0 to 44** | 13/757 (2%) | 16/377 (4%) | 0.02* |
|  | **45 to 64** | 39/521 (7%) | 24/253 (9%) | 0.33 |
|  | **65+** | 87/432 (20%) | 65/272 (24%) | 0.26 |
| **Deaths, stratified by the interval between TB and COVID-19 diagnoses** | **Within 90 days** | N/A | N/A | N/A |
|  | **Within 60 days** | N/A | N/A | N/A |
|  | **Within 30 days** | N/A | N/A | N/A |
| **Death was related to TB** | | Not assessed | 63/105 | N/A |

**+ Based on 322 and 230 individuals, respectively**

**++ Based on 1007 and 495 individuals, respectively**
