# Supplementary material for: Cohort study of the mortality among patients in New York City with tuberculosis and COVID-19, March 2020 to June 2022
Source: PLOS Glob Public Health. 2023 Apr 26;3(4):e0001758. doi: 10.1371/journal.pgph.0001758 (PMC10132536; doi:10.1371/journal.pgph.0001758)
Supplement: S3 Table — (DOCX) [file pgph.0001758.s005.docx]

S3 Table. Comparison of patients diagnosed with TB in NYC between 3/1/2020 and 6/30/2023 and not diagnosed with COVID-19 during that period (TB-alone group), versus patients diagnosed with both TB and COVID-19 during this period, but where the TB and COVID-19 diagnoses were over 120 days apart (non-concurrent).

| **Characteristic** | | **TB-alone**  **(n=902)** | **Non-concurrent TB/COVID-19 (n=133)** | **p-value** |
| --- | --- | --- | --- | --- |
| **Median [IQR] Age in Years at TB diagnosis** | | 51 [35, 68] | 56 [38, 69] | 0.13 |
| **Male sex** | | 583 (65%) | 72 (54%) | 0.02* |
| **US-born** | | 110 (12%) | 15 (11%) | 0.89 |
| **Race / Ethnicity (among US-born)** | |  |  | 0.22 |
| **Non-Hispanic White** | | 15 (14%) | 5 (4%) |  |
| **Non-Hispanic Black or**  **African American** | | 58 (53%) | 5 (4%) |  |
| **Hispanic** | | 25 (23%) | 3 (2%) |  |
| **Asian** | | 9 (8%) | 1 (1%) |  |
| **Other / Unknown** | | 3 (3%) | 1 (1%) |  |
| **Median [IQR] years living in US+** | | 12 [4, 25] | 15 [9, 27] | 0.06 |
| **Pulmonary involvement** | | 756 (84%) | 109 (82%) | 0.61 |
| **Cavitary Chest X-ray** | | 149 (20%) | 16 (12%) | 0.24 |
| **Ever Sputum Smear** **positive** | | 421 (56%) | 60 (55%) | 0.92 |
| **Multi-drug resistant (out of culture positive)** | | 12/758 (2%) | 5/116 (4%) | 0.06 |
| **History TB disease (documented or self-reported)** | | 54 (6%) | 6 (5%) | 0.69 |
| **Other health problems** | |  |  |  |
| **Diabetes** | | 218 (24%) | 40 (30%) | 0.16 |
| **HIV Status** | |  |  | 0.31 |
| **Infected** | | 40 (4%) | 3 (2%) |  |
| **Uninfected** | | 716 (79%) | 103 (77%) |  |
| **Unknown/refused** | | 146 (16%) | 27 (20%) |  |
| **Social risk factors within the past 12 months prior to diagnosis** | |  |  |  |
| **Homelessness** | | 37 (4%) | 0 (0%) | 0.01* |
| **Incarceration** | | 5 (1%) | 0 (0%) | 1.00 |
| **Injection drug use** | | 2 (0%) | 1 (1%) | 0.34 |
| **Non-injection drug use** | | 61 (7%) | 1 (1%) | 0.003** |
| **Alcohol abuse** | | 24 (3%) | 0 (0%) | 0.06 |
| **Smoked tobacco** | | 148 (16%) | 12 (9%) | 0.03* |
| **Median [IQR] days from cough onset to TB diagnosis ++** | | 50 [20, 93] | 58 [30, 98] | 0.28 |
| **Ever on Directly Observed Therapy for TB (among** **eligible)** | | 570/761 (75%) | 88/120 (73%) | 0.74 |
| **Hospitalized for TB** | | 464 (51%) | 69 (52%) | 1.00 |
| **Deaths** | | 105 (12%) | 9 (7%) | 0.10 |
| **Deaths prior to treatment initiation** | | 26/105 (25%) | 2/9 (22%) | 1.00 |
| **Deaths, stratified by age** | **0 to 44** | 16/377 (4%) | 0/45 (0%) | 0.39 |
|  | **45 to 64** | 24/253 (9%) | 3/40 (8%) | 1.00 |
|  | **65+** | 65/272 (24%) | 6/48 (13%) | 0.09 |
| **Deaths, stratified by the interval between TB and COVID-19 diagnoses** | **Within 90 days** | N/A | N/A |  |
|  | **Within 60 days** | N/A | N/A |  |
|  | **Within 30 days** | N/A | N/A |  |
| **Death was related to TB** | | 63/105 (60%) | 3/9 (33%) | 0.16 |

**+ Based on 230 and 34 individuals, respectively**

**++ Based on 495 and 74 individuals, respectively**
