# Supplementary material for: Cohort study of the mortality among patients in New York City with tuberculosis and COVID-19, March 2020 to June 2022
Source: PLOS Glob Public Health. 2023 Apr 26;3(4):e0001758. doi: 10.1371/journal.pgph.0001758 (PMC10132536; doi:10.1371/journal.pgph.0001758)
Supplement: Attachment — Submitted filename: ResponseToReviewers_27Feb2023.docx [file pgph.0001758.s006.docx]

Response to reviewers and editors

Journal Requirements:

1. Please insert an Ethics Statement at the beginning of your Methods section, under a subheading 'Ethics Statement'. It must include:

1) The name(s) of the Institutional Review Board(s) or Ethics Committee(s)

2) The approval number(s), or a statement that approval was granted by the named board(s)

3) (for human participants/donors) - A statement that formal consent was obtained (must state whether verbal/written) OR the reason consent was not obtained (e.g. anonymity). NOTE: If child participants, the statement must declare that formal consent was obtained from the parent/guardian.

**We have added to our ethics statement as follows:**

Ethics Statement

The New York City Department of Health and Mental Hygiene (NYC DOHMH) Institutional Review Board determined that this project (Protocol #20-061) was exempt pursuant to 45 CFR §46.104(d)(4)(iii). This project involves only data routinely collected as part of normal program services. Consent was not obtained for this secondary research analysis as the research activities only involved information that is regulated for public health purposes. This work was supported by NYC DOHMH Bureau of Tuberculosis Control program funds.

**This wording is in line with other PLOS publications where similar types of data were used, such as: Service utilization and HIV outcomes among transgender women receiving Ryan White Part A services in New York City (**[**https://doi.org/10.1371/journal.pone.0253444**](https://doi.org/10.1371/journal.pone.0253444)**); Novel Use of Surveillance Data to Detect HIV-Infected Persons with Sustained High Viral Load and Durable Virologic Suppression in New York City (**[**https://doi.org/10.1371/journal.pone.0029679**](https://doi.org/10.1371/journal.pone.0029679)**); Assessing Syndromic Surveillance of Cardiovascular Outcomes from Emergency Department Chief Complaint Data in New York City (**[**https://doi.org/10.1371/journal.pone.0014677**](https://doi.org/10.1371/journal.pone.0014677)**); Social and Genetic Networks of HIV-1 Transmission in New York City (**[**https://doi.org/10.1371/journal.ppat.1006000**](https://doi.org/10.1371/journal.ppat.1006000)**)**

2. Please provide separate figure files in .tif or .eps format only and remove any figures embedded in your manuscript file. Please also ensure that all files are under our size limit of 10MB.

For more information about figure files please see our guidelines:

<https://journals.plos.org/globalpublichealth/s/figures>
<https://journals.plos.org/globalpublichealth/s/figures#loc-file-requirement>

**Thank you. We have included .tif files that are under 10MB.**

3. We notice that your supplementary material are included in the manuscript file. Please remove them and upload them with the file type 'Supporting Information'. Please ensure that each Supporting Information file has a legend listed in the manuscript after the references list.

**Thank you. We removed the supplementary material from the manuscript file and uploaded them with the file type “Supporting Information”. We have ensured that each file has a legend in the manuscript after the references list.**

Additional Editor Comments (if provided):

Dear authors:

We have received the reviewers' comments. Additionally to them, consider these others:

1. Add the design to the title (STROBE guidelines), and to the methods section too.

**We have rephrased the title as:**

Cohort study of the mortality of patients in New York City with Tuberculosis and COVID-19, March 2020 to June 2022

**We modified the methods section of the abstract (lines 15-18):**

A retrospective cohort of patients who were diagnosed with TB and COVID-19 concurrently (within 120 days) between March 2020 and June 2022 in New York City (NYC) was identified. This cohort was compared with a cohort of patients diagnosed with TB-alone during the same period in terms of demographic information, clinical characteristics, and mortality.

**We also modified the methods section of the manuscript (lines 77-78):**

The demographic and clinical characteristics and mortality outcomes of retrospective cohorts of TB patients with and without COVID-19 in NYC were examined and compared.

**We also changed the language to replace some instances of “groups” with “cohorts” in this section. We do not refer to the COVID-19 comparison group as a cohort, because that comparison is based on limited information about each patient, and is not treated the same way as the two main cohorts of interest.**

2. The COVID-19 comparison group is not included in the objective of the study. You should clarify this. The same happens with comparisons performed with pre-pandemic patients. These patients are not part of the study period, either.

**We focused in more closely on the main particularly by revising the tables and figures in the manuscript (leaving information about the supplementary comparison groups in the supplement), and by changing some of the language (as shown below). We wish to keep information on the supplementary comparison groups available, though, for two reasons. First, we believe that including this information provides a transparent look at how we went about this project, and which cohorts we compared with each other, to make it clear that we did not cherry pick among possible comparison groups. Second, our objectives state that we aimed to compare mortality among patients with TB and COVID with patients with TB or COVID-19 alone. We focused on concurrent TB/COVID but wish to share results from non-concurrent TB/COVID as well, in the supplement. We also considered treating pre-pandemic as our TB-alone group but chose to focus on the 2020-2022 TB-alone population instead, as it was more comparable to the TB/COVID cohort in terms of demographic and clinical characteristics. Finally, while our access to information on patients with COVID-19 alone is limited, we believe that sharing the mortality figures for this population is a key part of meeting our objective.**

**Lines 119-125:**

The “TB-alone” dataset included patients diagnosed with TB in NYC between 3/1/2020 and 6/30/2022 who had never been diagnosed with COVID-19 as of 6/30/2022. To examine whether this cohort of patients was a typical representation of TB patients prior to the pandemic, a supplementary comparison cohort was also constructed, which only included patients who were diagnosed with TB between 2016 and 2018. This pre-pandemic cohort of patients is not the primary comparison cohort used here, as patients diagnosed with TB during the pandemic may have been different from patients diagnosed with TB pre-pandemic.

**Lines 128-134:**

The NYC Health Department’s dataset on COVID-19 cases and deaths in NYC was used as another supplementary comparison group. The version of this dataset published on 6/30/2022 was used to mirror the end of the study period.20 Similar to previous work from the NYC Health Department,14 raw case and death counts were used to calculate an overall crude case fatality rate for this population. This dataset included less information than was available for the main cohorts studied here and is only used as a point of reference for the crude case fatality rate for NYC residents diagnosed with COVID-19 during this period.

3. Calls to the figures must be made from the results section and not from the methods section. Please delete these calls in methods section.

**Calls to figures have been deleted from the methods section.**

4. Use the format of table 2 to redo table 1. Include statistics in all of the tables.

**To mirror the format of Table 2, we have transformed and compressed Table 1 and added a column for statistics. We considered inserting Table 1 into Table 2, but instead decided to have two tables with the same format; one on demographic and clinical variables and the other on outcomes. The numbers have changed due to the extension of the study period, though the conclusions remain similar. The information on the supplementary comparison groups has been moved into the supplementary tables. We have also swapped the order of Table 1 and Table 2. Thus, the old Table 1 is now Table 2, and looks like this:**

| **Characteristic** | | **TB-alone**  **(n=902)** | **Concurrent TB/COVID-19**  **(n=106)** | **p-value** |
| --- | --- | --- | --- | --- |
| **Hospitalized for TB** | | 464 (51%) | 57 (54%) | 0.68 |
| **Deaths** | | 105 (12%) | 23 (22%) | 0.005** |
| **Deaths, stratified by the interval between TB and COVID-19 diagnoses**+ | **Within 90 days** | “ | 20/84 (24%) | 0.003** |
|  | **Within 60 days** | “ | 18/66 (27%) | <0.001*** |
|  | **Within 30 days** | “ | 13/42 (31%) | 0.001** |
| **Deaths prior to treatment initiation** | | 26/105 (25%) | 9/23 (39%) | 0.20 |
| **Deaths, stratified by age** | **0 to 44** | 16/377 (4%) | 1/36 (3%) | 1.00 |
|  | **45 to 64** | 24/253 (9%) | 10/44 (23%) | 0.02* |
|  | **65+** | 65/272 (24%) | 12/26 (46%) | 0.02* |
| **Death was related to TB** | | 63/105 (60%) | 13/23 (57%) | 0.82 |

+ As a sensitivity check on whether the mortality comparisons are affected by the definition of concurrent TB/COVID-19 as within 120 days, analyses were also done where concurrent TB/COVID-19 was defined by diagnoses within shorter periods of days. All comparisons are with the TB-alone group (n=902).

Fisher Exact tests were used to calculate p-values. Significance levels: * p < 0.05, ** p <0.01, *** p<0.001.

5. Statistics used should be mentioned at tables footer (not in the columns titles). They should be detailed for each variable.

**The names of statistical tests have now been moved to the table footers, and removed from the column titles. P-values are now shown and described in each table.**

6. It is note clear why didn't you control other confounders in the multivariable model. This should be cleared.

**When building the regression model, we controlled for variables that we expected to be important based on existing literature on TB and COVID mortality as well as variables that were associated with death versus survival in patients with concurrent TB/COVID (as shown in Table 3) or that differed between patients with TB-alone and concurrent TB/COVID-19 (Table 1). For these reasons, we chose to control for age, sex and diabetes. No variables examined here besides age, sex and diabetes were significantly different when comparing TB/COVID-19 versus TB-alone patients, or when comparing TB/COVID-19 patients who died versus those who survived. Furthermore, some variables had low variability or a high number of missing values.**

**Finally, we did not include diabetes in the adjusted model because diabetes and age exhibited collinearity. We chose to include age over diabetes because age could be a proxy for a range of health risks, including diabetes. Since many readers may be curious about the impact of diabetes on mortality in this patient population, we also did a subset analysis where we ran the regression separately for patients with diabetes and without diabetes. This subset analysis is shown in the regression output in the supplementary materials and referenced in the added text shown below.**

**As an additional sensitivity check, we tried running the regression controlling for every single variable listed in Tables 1 and 3, adding one at a time to the model where we already controlled for age and sex. We did not find any variable that meaningfully changed the hazard ratio in terms of magnitude or significance. Thus, our findings of a hazard ratio of ~2 are robust to the inclusion of additional covariates which did not meet our criteria for inclusion.**

**We have added to the methods and results, as follows:**

**Lines 152-162 (methods):**

Cox proportional hazards regression was used to assess the hazard of death past the date of TB diagnosis for patients with concurrent TB/COVID-19 patients versus TB-alone. Time-to-event was defined as the number of days between TB diagnosis and reported death date. Variables were included in the partially adjusted model only if they were either a) significantly different in the bivariate analyses comparing patients with concurrent TB/COVID-19 who died versus those who survived or comparing patients with concurrent TB/COVID-19 versus TB-alone, or b) known to be important predictors of COVID-19 mortality. Controlling for age,^27, 28^ sex^29, 30^ and diabetes^31, 32^ were prioritized because these variables are known to be associated with COVID-19 mortality and are collected in a highly standardized manner in the TB electronic disease surveillance and case management system. ~~We considered controlling for diabetes as well, because diabetes is also known to be associated with COVID-19 mortality31, 32 and data on diabetes is also collected in a highly standardized manner. However, since age and diabetes were correlated, the results controlling for both age and diabetes are only shown in the supplementary materials.~~ Variables were excluded from the model if they were colinear with other variables in the model.

**Lines 219-228 (results):**

Multivariable Cox proportional hazards regression suggests that the hazard of death is 2.62 (CI 1.66-4.13, p<0.001) times higher for patients with concurrent TB/COVID-19 when compared to patients with TB-alone after controlling for age and sex. ~~The results are similar if diabetes is controlled for as well, but~~ since age and diabetes are correlated (R2=0.12, p<0.001 for the 1141 patients diagnosed with TB between 3/1/2020 and 6/30/2022), this model was not adjusted for diabetes ~~the results are only shown controlling for age (all results are shown in the supplementary materials)~~. As a sensitivity check, when patients are subsetted into those with and without diabetes, the results of this regression were similar (supplementary materials). No other variables were significantly different in the bivariate analyses comparing patients with concurrent TB/COVID-19 who died versus those who survived (Table 3) or between patients with TB-alone versus concurrent TB/COVID-19 (Table 1), so no additional variables were included in this partially adjusted regression model.

7. Table 4 should show results of the crude and adjusted analysis for all of the variables included in the multivariable analysis.

**We decided that it would make the most sense to remove Table 4, since we could quickly summarize its findings in the text, as shown below. We describe above (in the response to point #6) why our methodology led us to control for age and sex.**

**The text describing the findings of what was previously Table 4 is as follows:**

Multivariable Cox proportional hazards regression suggests that the hazard of death is 2.62 (CI: 1.66-4.13, p<0.001) times higher for patients with concurrent TB/COVID-19 when compared to patients with TB-alone after controlling for age and sex. Since age and diabetes are correlated (R2=0.12, p<0.001 for the 1141 patients diagnosed with TB between 3/1/2020 and 6/30/2022), this model was not adjusted for diabetes. As a sensitivity check, when patients are subsetted into those with and without diabetes, the results of this regression were similar (supplementary materials). No other variables were significantly different in the bivariate analyses comparing patients with concurrent TB/COVID-19 who died versus those who survived (Table 3) or between patients with TB-alone versus concurrent TB/COVID-19 (Table 1), so no additional variables were included in this partially adjusted regression model. The unadjusted hazard of death was similar at 2.18 (CI: 1.39-3.42, p<0.001). These regressions were based on 1008 individuals (902 with TB-alone and 106 with concurrent TB/COVID-19) and 128 events (105 deaths among patients with TB-alone and 23 among patients with concurrent TB/COVID-19).

8. Double check the use of the word "multivariate" instead of "multivariable".

**We have corrected the word “multivariate” to be “multivariable” in line 219.**

9. Include in the methods section the description of the time-to-event variable used to perform the survival multivariable analysis.

**Thank you. We have added the following to the methods (lines 153-155):**

Cox proportional hazards regression was used to compare mortality between patient cohorts. Time-to-event was defined as the number of days between TB diagnosis and reported death date or 30 days after treatment completion.

10. The full name of NYC DOHMH should be mentioned at Ethics section.

**Thank you, we have written out “New York City Department of Health and Mental Hygiene (NYC DOHMH)” in the ethics section, before subsequently using the acronym.**

11. The meaning of dots and lines is not clear in Figure 1. There are more combinations of "colors and lines" than are named in the legends. This should be reviewed

**To clarify, we have added the following. We have also moved part B of this figure into the supplement, so that legend is shown below as well.**

Fig 1. Timeline of diagnoses for concurrent TB/COVID-19 patients diagnosed with TB between 3/1/2020 and 6/30/2022 (n=106).

The dates when patients were diagnosed with TB ~~diagnoses~~ are shown by blue points and the dates when patients were diagnosed with COVID-19 ~~diagnoses~~ are shown by yellow points. The line between points represents the time ~~interval~~ between diagnosis of the two diseases. The line is the color of the first diagnosis; thus, in cases where COVID-19 was diagnosed first, the line is yellow, and in cases where TB was diagnosed first, the line is blue. Deaths are shown ~~in~~ as red points. Only patients with concurrent TB/COVID-19 ~~patients~~ are shown above (n=~~44~~ 106). Patients with ~~and~~ non-concurrent TB/COVID-19 ~~patients~~ are shown in Figure S1 (n=~~34~~ 133). ~~Ten~~ Twenty-three deaths occurred in the concurrent group and ~~one~~ nine deaths occurred in the non-concurrent group.

Fig S1. Timeline of diagnoses for non-concurrent TB/COVID-19 patients diagnosed with TB between 3/1/2020 and 6/30/2022.

The dates when patients were diagnosed with TB are shown by blue points and the dates when patients were diagnosed with COVID-19 are shown by yellow points. The line between points represents the time between diagnosis of the two diseases. The line is the color of the first diagnosis. Deaths are shown as red points. Nine deaths occurred among these patients; however, on death occurred after 6/30/2022, but before that patient completed treatment for TB, and is thus not visible here. Five deaths occurred during a COVID wave in late 2021. Four of these deaths occurred in a hospital, and COVID was not noted in their death certificates. The fifth person who died was diagnosed with COVID soon after their death. Thus, probably only one of these five deaths during this COVID wave was due to COVID, since it is likely that COVID infection would not have been missed in a hospitalized patient in December 2021.

12. Figure 2 is redundant, given that the same information is at Table 1. Consider to delete it.

**We agree and have thus removed this figure from our submission.**

13. Consider to delete "Investigation of supplementary comparison populations" at supplementary section, given that is not related with the objective of the study

**Thank you. We have removed this supplement, as we agree that is it discusses issues that are not related closely enough to our stated objectives. However, we retained a set of supplementary tables, which mirror the tables in the manuscript. We believe that these supplementary tables will be of interest to some readers, if they have follow-up questions when they see our primary analyses. We believe that mentioning the supplementary comparison groups briefly in the body of the text and having them available in the supplement provides the reader a fuller picture of what we did. We condensed the discussion of the supplementary comparison groups to a single section in the results:**

Mortality among supplementary comparison groups

When compared with patients with TB-alone, a similar percentage of patients with non-concurrent TB/COVID-19 died (Table S1). However, a lower percentage of patients with COVID-19 alone and TB diagnosed pre-pandemic died (Table S2, Table S3).

14. The use of third person pronouns is preferred ("it" instead of "we" or "I")

**Use of the pronouns we and I have largely been replaced with third person pronouns. In a small number of cases, they were kept where it was very difficult to replace them without changing the meaning of the text.**

Please include/answer each one of all these comments.


[Note: HTML markup is below. Please do not edit.]

Reviewers' comments:

Reviewer's Responses to Questions

**Comments to the Author**

1. Does this manuscript meet PLOS Global Public Health’s [publication criteria](https://journals.plos.org/globalpublichealth/s/criteria-for-publication)? Is the manuscript technically sound, and do the data support the conclusions? The manuscript must describe methodologically and ethically rigorous research with conclusions that are appropriately drawn based on the data presented.

Reviewer #1: Yes

Reviewer #2: Partly

2. Has the statistical analysis been performed appropriately and rigorously?

Reviewer #1: Yes

Reviewer #2: No

3. Have the authors made all data underlying the findings in their manuscript fully available (please refer to the Data Availability Statement at the start of the manuscript PDF file)?

The [PLOS Data policy](https://journals.plos.org/globalpublichealth/s/data-availability) requires authors to make all data underlying the findings described in their manuscript fully available without restriction, with rare exception. The data should be provided as part of the manuscript or its supporting information, or deposited to a public repository. For example, in addition to summary statistics, the data points behind means, medians and variance measures should be available. If there are restrictions on publicly sharing data—e.g. participant privacy or use of data from a third party—those must be specified.

Reviewer #1: Yes

Reviewer #2: Yes

4. Is the manuscript presented in an intelligible fashion and written in standard English?

PLOS Global Public Health does not copyedit accepted manuscripts, so the language in submitted articles must be clear, correct, and unambiguous. Any typographical or grammatical errors should be corrected at revision, so please note any specific errors here.

Reviewer #1: Yes

Reviewer #2: Yes

5. Review Comments to the Author

Please use the space provided to explain your answers to the questions above. You may also include additional comments for the author, including concerns about dual publication, research ethics, or publication ethics. (Please upload your review as an attachment if it exceeds 20,000 characters)

Reviewer #1: Excellent and much-needed analysis of mortality among people infected with both Covid and TB.

The authors have clearly listed the limitations, including the small number of deaths, inadequate adjustment of confounding, and undiagnosed Covid during the study period.

I have a few suggestions for improvement:

1. Since the study period is mostly during the first year of the pandemic, and before widespread access to vaccination, it might be good to explicitly mention this in the title (e.g. Mortality among patients in New York City with Tuberculosis and COVID-19 during the first year of the pandemic, or something like that).

**Thank you, we solved this problem in two ways. First, we have extended the time frame, and second, we rephrased the title as:**

Cohort study of the mortality of patients in New York City with Tuberculosis and COVID-19, March 2020 to June 2022

2. The study validity rests a lot on the two databases: NYC TB electronic disease surveillance and case management system, and the communicable disease surveillance system in NYC. The authors need to do a better job of explaining these systems and the quality of data they provide. How complete is the reporting in these databases, and how easy is it to cross-match or do the records linkage? What unique ID is used to do the linkage and in what % of the cases was it difficult to link? What is the duration of follow-up of patients in the databases? Are there any published studies on the quality of data in these two systems?

**We have added to the methods as follows to provide more information about these databases and the potential for missed matches. The US does not have a system of universal ID numbers, though the social security number comes close. However, not all individuals in the US are required to have a social security number, and many individuals may be hesitant to give their social security number on forms. Thus, the best technique available to us was to do a deterministic match based on demographic identifiers including name, date of birth, social security number, phone number and address (as stated in lines 103-108 in the methods). We have no perfect way to know how many matches were missed, as many people show up in one database and not the other, and this may be due to them only having been diagnosed with one disease and not the other. We have added some information to describe the case definitions used for both diseases, how TB data quality is maintained, and where the reader can find more published and online information on the COVID dataset.**

Demographic and clinical information on patients with TB in NYC was drawn from the NYC TB electronic disease surveillance and case management system (Maven 5.4., Conduent Inc., Florham Park, NJ). All patients had confirmed TB disease and were reported to the CDC as a Verified Case of TB. TB is a mandatory reportable condition in the United States, which is required to be reported within 24 hours by both providers and laboratories, resulting in high reporting completeness. Demographic and clinical variables were collected by case managers during routine chart abstractions and interviews and entered into the TB surveillance and case management system. Additional information in this database is reported by laboratories and clinical providers. Information on deaths from any cause among patients with TB is collected by case managers and confirmed by obtaining death certificates with subsequent medical review. Chart reviews for patients are routine, intense and repeated. Patients are followed from the time their disease is reported to the NYC Health Department until treatment completion, death, or loss to follow-up after repeated efforts to return a patient to care. Data is entered and updated through the course of treatment.

Data on COVID-19 diagnoses and related deaths were obtained from the NYC communicable disease surveillance system (Maven 5.5, Conduent Inc., Florham Park, NJ). The case definitions for COVID-19 and TB are those used by the Council of State and Territorial Epidemiologists.18, 19 Data on COVID-19 cases and deaths in NYC are available publicly for download, alongside data visualizations and in-depth variable definitions.15, 20, 21

**We have also added the following to the limitations section of the discussion:**

TB data quality is high due to intensive case management, but the match between the TB and COVID-19 datasets is also reliant on accurately reported demographics in the COVID-19 database. This depends on accurate COVID-19 test reporting by laboratories. Missed matches could lead to an underestimate of the number of patients with TB/COVID-19.

If any of the deaths that occurred in patients in the TB-alone cohort were due to undiagnosed COVID-19 around the time of their death, or to COVID-19 laboratory reporting data quality issues, this could have artificially reduced our measurement of the impact of COVID-19 on mortality in patients with TB.

3. The hazard ratios provided must be viewed as partially adjusted, as a full adjustment of potential confounders could not be done. So, please explicitly state this.

**We now refer to this model as partially adjusted, as you recommend. We have described in our response to Reviewer #1’s 6th point why we chose not to adjust for a wider range of potential confounders. We have added the following to the text to clarify this point for the reader.**

**Lines 155-159 (methods):**

Variables were included in the partially adjusted model only if they were either a) significantly different in the bivariate analyses comparing patients with concurrent TB/COVID-19 who died versus those who survived or comparing patients with concurrent TB/COVID-19 versus TB-alone, or b) known to be important predictors of COVID-19 mortality.

**Lines 218-224 (results):**

Multivariable Cox proportional hazards regression suggests that the hazard of death is 2.62 (CI: 1.66-4.13, p<0.001) times higher for patients with concurrent TB/COVID-19 when compared to patients with TB-alone after controlling for age and sex. Since age and diabetes are correlated (R2=0.12, p<0.001 for the 1141 patients diagnosed with TB between 3/1/2020 and 6/30/2022), this model was not adjusted for diabetes. As a sensitivity check, when patients are subsetted into those with and without diabetes, the results of this regression were similar (supplementary materials). No other variables were significantly different in the bivariate analyses comparing patients with concurrent TB/COVID-19 who died versus those who survived (Table 3) or between patients with TB-alone versus concurrent TB/COVID-19 (Table 1), so no additional variables were included in this partially adjusted regression model.

4. It will be good for the authors to expand the time period and do a larger study by included patients during 2021 and 2022, acknowledging the challenges in accounting for vaccination rates and testing rates that may have changed over time. Access to paxlovid might be another confounding variable. What should others who plan similar studies in future worry about in terms of design and analysis? Kindly provide some suggestions for additional research.

**Thank you. We have been able to expand the study period through 6/30/2022. We chose this cutoff (rather than, for example, 12/31/2022) to allow for sufficient time for case managers to update patient files, for at least 6 months of follow-up for everyone diagnosed with TB, and for some time to identify most deaths that occurred. This means that our dataset now covers a range of situations in terms of how COVID was treated in NYC—including when COVID was under-diagnosed because tests weren’t available, through when COVID was under-reported because many people had access to antigen tests at home. We have added some language to the limitations section, shown below, to describe this situation. We do not have information in the TB database on how each patient was treated for COVID, or whether they were vaccinated for COVID. The fact that deaths occurred throughout the study period, and that the elevated frequency of death remained significant even once the study period was extended to include a period of time after many peoples’ lives went “back to normal” suggests that the risk to patients with concurrent TB/COVID was not limited to the first year of the COVID-19 pandemic.**

**The change in the study period necessitated changes to the limitations section of the manuscript. We also discuss some future work that could be beneficial once these hurdles can be overcome:**

Limitations

A relatively small sample size and limited period of follow-up time prevented us from thoroughly examining the impact of a wider range of potential confounding demographic and clinical variables on patient outcomes. Furthermore, the data available for each patient was limited to what is routinely collected for patients with TB in NYC, and thus did not include information on the severity of COVID-19 disease, or the treatments received during an episode of COVID-19.

While the availability of disease and mortality data for both TB and COVID-19 is a strength of this study, it is likely that many cases of COVID-19 went undiagnosed in NYC during the study period—including in some patients with TB. Early on, access to tests for COVID-19 was severely limited, leading to test positivity rates that peaked at 69% on 3/30/2020.15 Later, the use of at-home tests likely led to a rise in unreported COVID-19 cases.37 While many hospital-based clinicians ordered COVID-19 tests for patients during their workup for TB, and clinicians in TB clinics often recommended getting COVID-19 tests to patients experiencing symptoms, COVID-19 was not systematically ruled out as part of the care and monitoring of the patients with TB included in this study.

TB data quality is high due to intensive case management, but the match between the TB and COVID-19 datasets is also reliant on accurately reported demographics in the COVID-19 database. This depends on accurate COVID-19 test reporting by laboratories. Missed matches could lead to an underestimate of the number of patients with TB/COVID-19.

If any of the deaths that occurred in patients in the TB-alone cohort were due to undiagnosed COVID-19 around the time of their death, or to COVID-19 laboratory reporting data quality issues, this could have artificially reduced our measurement of the impact of COVID-19 on mortality in patients with TB.

Future work

~~This study represents the impact of infection with SARS-CoV-2 variants that are no longer the dominant variants in NYC, in a population of New Yorkers where COVID-19 vaccination was less common than it is currently.~~ The level of mortality that results from concurrent TB/COVID-19 in the future will depend both on risk mitigation tools available to each patient (including vaccination and treatment options) as well as the risk profile of the SARS-CoV-2 variant that has infected each patient. ~~Thus, our results represent what happened to TB/COVID-19 patients in NYC before Delta and subsequent variants became common, and before vaccination became more widespread in NYC.~~

We will continue to see additional patients with concurrent TB/COVID-19. It will remain important to critically assess short and long-term outcomes for these patients. Future studies from broader geographies and covering a longer period will enable the examination of case fatality rates for patients with TB/COVID-19 over time and in comparison with other patient populations. Future work could also examine the mortality of patients who are diagnosed with COVID-19 at any time when they are being treated for TB, or after they have been cured of TB. As COVID-19 surveillance is incorporated into standard practices in health departments around the US, it will hopefully become easier to integrate datasets from different agencies and jurisdictions.

5. I agree with the recommendation about giving priority to people with TB for early access to Covid vaccination and would also add that they should be prioritized for easy access to anti-virals like paxlovid.

**Thank you. We have added this to the conclusions, as follows:**

Given the growing recognition of the risks posed by these diseases in combination, it will be important to increase screening for COVID-19 among patients with TB and increase screening for TB among patients hospitalized with suspected COVID-19. Early detection of COVID-19 in patients with TB should then be followed by guidance towards easy and immediate access to anti-viral medications, and any other effective medications for COVID-19 that are developed in the future. In DOHMH TB clinics in NYC, this means that providers can refer patients with COVID-19 symptoms to co-located COVID-19 testing sites, and staff regularly refer patients with TB to sites where they can be vaccinated against COVID. Treating clinicians will also need to have access to information regarding interactions between anti-TB medications and COVID-19 treatments. These findings highlight the importance of making sure that TB is recognized as an important comorbidity for patients with COVID-19, and vice versa. Wide recognition among clinicians, public health agencies and TB patients of the risks posed to patients diagnosed with concurrent TB and COVID-19 could help ensure that the risks are mitigated for these patients.

6. During the study period, how easy was it for New Yorkers to access Covid testing? what was the testing rate and positivity rate in NYC? Can you please cite data on this? If it was very difficult, then it would likely mean that Covid was under-diagnosed in the entire population (which also include those with TB).

**We can provide additional context on this topic, using the same public dataset which was used to generate the supplementary comparison group of COVID-alone patients. We have added the following text to the limitations. Of course, the context has now changed a bit since we have expanded the timeframe of the study. These additions were also shown above as part of our response to your 4^th^ point.**

While the availability of disease and mortality data for both TB and COVID-19 is a strength of this study, it is likely that many cases of COVID-19 went undiagnosed in NYC during the study period—including in some patients with TB. Early on, access to tests for COVID-19 was severely limited, leading to test positivity rates that peaked at 69% on 3/30/2020.15 Later, the use of at-home tests likely led to a rise in unreported COVID-19 cases.37 While many hospital-based clinicians ordered COVID-19 tests for patients during their workup for TB, and clinicians in TB clinics often recommended getting COVID-19 tests to patients experiencing symptoms, COVID-19 was not systematically ruled out as part of the care and monitoring of the patients with TB included in this study.

TB data quality is high due to intensive case management, but the match between the TB and COVID-19 datasets is also reliant on accurately reported demographics in the COVID-19 database. This depends on accurate COVID-19 test reporting by laboratories. Missed matches could lead to an underestimate of the number of patients with TB/COVID-19.

If any of the deaths that occurred in patients in the TB-alone cohort were due to undiagnosed COVID-19 around the time of their death, or to COVID-19 laboratory reporting data quality issues, this could have artificially reduced our measurement of the impact of COVID-19 on mortality in patients with TB.

Reviewer #2: The manuscript aims to describe the mortality amongst TB/COVID-19 patients in comparison with patients with TB only (or COVID-19 only) during the period March 2020-May 2021 in NYC. The topic is interesting, although already addressed by similar papers in different settings. However, presented data do not add much to the available knowledge and would benefit from major re-writing plus addition of details before being considered for publication. Specific comments are provided here below for authors' consideration.

1. Abstract is confusing as it contains results under method section (e.g. "We identified 44 patients who were diagnosed with TB and COVID-19..... compared to those diagnosed with TB-alone during the same period (n=421)". Please edit the method and results section of the abstract accordingly.

**Thank you. We have rearranged the text as follows:**

Methods:

A retrospective cohort of patients who were diagnosed with TB and COVID-19 concurrently (within 120 days) between March 2020 and June 2022 in New York City (NYC) was identified. This cohort was compared with a cohort of patients diagnosed with TB-alone during the same period in terms of demographic information, clinical characteristics, and mortality. Cox proportional hazards regression was used to compare mortality between patient cohorts.

Results:

One hundred and six patients with concurrent TB/COVID-19 were identified and compared with 902 patients with TB-alone. These two cohorts of patients were largely demographically and clinically similar. However, mortality was higher among patients with concurrent TB/COVID-19 in comparison to patients with TB-alone, even after controlling for age and sex (hazard ratio 2.62, 95% Confidence Interval 1.66-4.13). Nearly one in three (22/70, 31%) patients with concurrent TB/COVID-19 aged 45 and above died during the study period.

2. How were COVID-19 and TB diagnosis formulated' Please specify (Authors mention that both confirmed and probable COVID-19 patients were included but not details are provided). How many were confirmed TB cases? How many P/EP? Radiological findings (i.e. how many had extensive CXR abnormalities)? Some data are included in tables but not mentioned in the text. Please add as they may play a role in determining case severity and, hence, risk of death.

**The case definitions are standardized in the US by the CSTE, and all TB cases were confirmed and included in national CDC counts as a Report of a Verified Case of TB. We have added the following to clarify the surveillance case definitions for COVID-19 and TB:**

Data sources

Line 87: All patients had confirmed TB disease and were reported to the CDC as a Verified Case of TB.

Line 101: The case definitions for COVID-19 and TB are those used by the Council of State and Territorial Epidemiologists.18, 19 Data on COVID-19 cases and deaths in NYC are available publicly for download, alongside data visualizations and in-depth variable definitions.15, 20, 21

Line 111: The match was based on demographic identifiers including name, date of birth, social security number, phone number and address. Cases of both confirmed and probable COVID-19 were included.15, 20

**Regarding your point about case severity: in Table 1, we report that 756 (84%) of patients with TB-alone and 86 (81%) patients with concurrent TB/COVID-19 had TB with pulmonary involvement. Among these individuals, a cavitary chest X-ray was recorded for 149/756 (20%) in the TB-alone group and 13/86 (81%) in the concurrent TB/COVID-19 group. We also include sputum smear positivity and the number of patients with MDR TB, as additional variables that are important for case severity for TB. Our relatively small sample size may have limited our ability to detect differences in these variables in Table 1 and Table 3. The way data is collected for public health purposes in our jurisdiction also limits our ability to examine additional variables that might be indicators of TB disease severity. We have emphasized these findings in the results, and this limitation in the conclusions, as follows:**

**Addition to results:**

We did not find significant differences between patients with concurrent TB/COVID-19 versus patients with TB-alone in terms of age, ~~sex,~~ whether the patient was US-born, race/ethnicity among US-born patients, TB disease site, whether the patient ever had a cavitary chest X-ray, whether the patient was ever sputum smear positive, HIV status, social risk factors for TB, and whether the patient had MDR-TB (Table 1).

**Addition to conclusions:**

Even after doing sensitivity checks on how we defined “concurrent” TB/COVID-19 (as diagnoses within 120, 90, 60, or 30 days) and controlling for patient age and sex, ~~we found that~~ death rates were significantly higher among patients with concurrent TB/COVID-19 than among patients with TB-alone. It is surprising that the percentage of patients with concurrent TB/COVID-19 who died was so high, given that the period covered included not only the “lockdown” period in NYC, but also 18 months in 2021 and 2022 in which vaccination and access to anti-viral and other treatments became increasingly widespread.

When comparing patients with concurrent TB/COVID-19 who died versus those who survived, the only significant differences identified were that patients who died were more likely to be older and more likely to have diabetes. In previous work, it was found that among both US-born and non-US-born patients with TB, TB-related death was associated with increased age, being culture positive and having both pulmonary and extra-pulmonary disease, though additional demographic and social risk factors were associated with TB-related death for non-US-born patients specifically.35

*Limitations*

A relatively small sample size and limited period of follow-up time prevented us from thoroughly examining the impact of a wider range of potential confounding demographic and clinical variables on patient outcomes. Furthermore, the data available for each patient was limited to what is routinely collected for patients with TB in NYC, and thus did not include information on the severity of COVID-19 disease, or the treatments received during an episode of COVID-19.

3. How many patients died during intensive phase of TB-treatment and how many during continuation phase? Pleas add.

**We think this is interesting, particularly in terms of whether deaths occurred while patients were acutely sick with TB, or once they were already well on their way towards being cured of TB. However, splitting deaths between those that occurred during the intensive versus continuation phase is difficult because there was a lot of variation in how patients were treated. For example, some patients had interruptions in their TB treatment and were restarted on an intensive phase, were started on an MDR regimen, were started on RIPE but changed to a different regimen due to resistance or intolerance, etc. Additionally, the definition of concurrent TB/COVID meant that most patients were diagnosed with both diseases around the same time, limiting the number of patients who died late in their treatment course. Thus, we have responded to this point by adding into the table the number of patients who died prior to treatment initiation versus during treatment. This is now in Table 2. As you recommended below, we have excluded any deaths that occurred more than 30 days after the end of TB treatment completion. We grouped deaths that occurred during treatment and those that occurred within 30 days of treatment completion because of some of the ambiguity about when treatment is officially considered to be complete. For example, if a patient has an expected date of completion on June 1, but died on May 15, it is possible that the medical team will decide to categorize the patient as having completed sufficient treatment, even if the patient would have been treated for a couple of extra weeks if they had lived. A line has been added to the methods to clarify this:**

Deaths were only included if they occurred prior to 30 days after the successful completion of treatment for TB disease. The 30-day window was used in order to avoid excluding patients whose treatment was administratively closed due to their death or imminent death, even though treatment might have continued if the patient had survived.

**Line added to outcomes tables (tables 2, S1 and S2):**

Deaths prior to treatment initiation

**These results are described in the results as follows (lines 195-198):**

Among patients with concurrent TB/COVID-19 and TB-alone, similar percentages of deaths were classified as related to TB by DOHMH physicians based on death certificates (57% and 60%, p=0.82), and similar percentages of patients were hospitalized for TB (54% and 51%, p=0.68, Table 2).

4. In TB-alone patients, was COVID-19 ruled out? If so, how?

**COVID-19 was ruled out for some patients, but not for all. If patients were hospitalized, the majority would have been tested, except perhaps in March 2020, when tests were in short supply. While many TB patients are diagnosed in hospitals, some were diagnosed in our clinics, where COVID-19 testing was not available at first. Later on, some of our TB clinics in NYC also had COVID-19 testing in the same building. However, it was up to the patients with TB to decide if they wanted to get a COVID test (it was not a requirement to get tested for COVID before being seen for their TB care) and not all patients chose to get a test around the time they were diagnosed with TB.**

**We have added the following line to the methods (the first additions are in response to other points made by both reviewers):**

While the availability of disease and mortality data for both TB and COVID-19 is a strength of this study, it is likely that many cases of COVID-19 went undiagnosed in NYC during the study period—including in some patients with TB. Early on, access to tests for COVID-19 was severely limited, leading to test positivity rates that peaked at 69% on 3/30/2020.15 Later, the use of at-home tests likely led to a rise in unreported COVID-19 cases.37 While many hospital-based clinicians ordered COVID-19 tests for patients during their workup for TB, and clinicians in TB clinics often recommended getting COVID-19 tests to patients experiencing symptoms, COVID-19 was not systematically ruled out as part of the care and monitoring of the patients with TB included in this study.

TB data quality is high due to intensive case management, but the match between the TB and COVID-19 datasets is also reliant on accurately reported demographics in the COVID-19 database. This depends on accurate COVID-19 test reporting by laboratories. Missed matches could lead to an underestimate of the number of patients with TB/COVID-19.

If any of the deaths that occurred in patients in the TB-alone cohort were due to undiagnosed COVID-19 around the time of their death, or to COVID-19 laboratory reporting data quality issues, this could have artificially reduced our measurement of the impact of COVID-19 on mortality in patients with TB.

5. How many patients were hospitalized? How many in ICU?

**We have information on which patients were hospitalized for TB, and have added this to Table 2 and Table 3, as well as Table S1 and Table S2. The additional line in Table 2 and Table 3 are as follows:**

| **Characteristic** | **TB-alone**  **(n=902)** | **Concurrent TB/COVID-19**  **(n=106)** | **p-value** |
| --- | --- | --- | --- |
| **Hospitalized for TB** | 464 (51%) | 57 (54%) | 0.68 |

| **Characteristic** | **Died (n=23)** | **Survived (n=83)** | **p-value** |
| --- | --- | --- | --- |
| **Hospitalized for TB** | 11 (49%) | 46 (55%) | 0.64 |

**However, information on which patients were hospitalized or admitted to the ICU for COVID is not collected as part of routine data collection as part of the normal program services of the NYC Department of Health’s Bureau of TB Control. While information on hospitalization and ICU admission was collected from a sample of patients, for example by some hospital networks in NYC^1^ and many hospitals around the world,^2^ this was not information that was available to us for the patients in our cohorts. Furthermore, we believe that this information was collected in a way that changed over time and was limited to subsamples of patients. Thus, it is not possible for us to include this information in our analyses. We have acknowledged this deficit as follows in the limitations:**

A relatively small sample size and limited period of follow-up time prevented us from thoroughly examining the impact of a wider range of potential confounding demographic and clinical variables on patient outcomes. Furthermore, the data available for each patient was limited to what is routinely collected for patients with TB in NYC, and thus did not include information on the severity of COVID-19 disease, or the treatments received during an episode of COVID-19.

6. How many patients did need oxigen-therapy? How many needed high-flow ventilation? Please disaggregate the mortality among different groups

**Please see response above to #5.**

7. Which treatment did COVID-19 patients receive? Anti-viral? Immunusuppressant? Steroids? Please specify.

**Please see response above to #5.**

8. Ref. 13 should be corrected as authors go under the name: "TB/COVID-19 Global Study Group"

**We apologize for not noticing this Endnote error. We have now fixed the way this reference prints.**

9. Statistical analysis should be included in table 1 (and mentioned into the text). Table 2 should be the first table presented as it describes the study population characteristics.

**Thank you, this was also suggested by the editor. We have reformatted Table 1 and added statistical analyses, as shown in the response to the editor’s 4^th^ point. Additionally, we have swapped the order of Table 1 and Table 2.**

10. Mortality is on the high side not only for TB/COVID19 patients but also for TB-alone patients (reaching 9% of total patients, with 21% in the 65+ age band in the study period and 8% in pre-pandemic time). Can authors comment on this aspect which is really worrying and propose corrective actions (i.e. COVID-19 pandemic does not seem to be an explanation for the high number of death, according to supplementary material, so there should be other factors to explain this, please elaborate on this point)? Deaths after completion of TB treatment should be counted as related to TB (if the treatment was successfully completed - so please correct 144 deaths of pre-pandemic period to 138)

**Thank you for this comment. Your interest in this question is why we chose to include information on mortality in the pre-pandemic TB cohort in the supplementary material (Table S2). We agree that the mortality among patients ages 65 and above with TB is distressingly high in all cohorts. It’s even higher when we extend the timeline out to 6/30/2022. We have commented on this here. We have also removed the confidence intervals, because the percentages shared are not estimates—they are the raw percentage of individuals who died. The confidence intervals were included previously to match the figure, but that figure has now been removed on the recommendation of Reviewer #1.**

Among patients 65 years and above, 46 ~~42~~% ~~(confidence interval [CI]: 19.3%-68.0%)~~ of patients with concurrent TB/COVID-19 died while 24 ~~17~~% ~~(CI: 16.8-17.3%)~~ of COVID-19 patients died and 24 ~~21~~% ~~(CI: 14.7-29.0%)~~ of TB-alone patients died (Table 2~~1~~, ~~Fig 2~~ Table S3). The number of deaths seen here is distressingly high; this was also true for TB patients prior to the pandemic, though the overall death rate was lower pre-pandemic (8% pre-pandemic versus 12% in the TB-alone group, Table S1). This is similar to the 8.7% of patients with TB diagnosed in NYC between 2004 and 2013 who died from any cause.31

**We have noted your concern that the deaths that occurred after the completion of treatment for TB should not be counted among the deaths listed in the tables. We have removed all deaths that occurred more than 30 days after completing treatment for TB. We have added this to the methods section as shown below.**

Deaths were only included if they occurred prior to 30 days after the successful completion of treatment for TB disease. The 30-day window was used to avoid excluding patients whose treatment was administratively closed due to their death or imminent death, even though treatment might have continued if the patient had survived.

11. Last but not least, it would be highly relevant to run similar analysis (i.e. comparison betwen mortality in TB/COVID-19 and TB-alone patients) including recent data, to understand if the recent approach to COVID-19, including vaccination, played a role in reducing mortality in TB/COVID-19 patients compared to the first phase of the pandemic. This last point would add very interesting information that would position this paper as a priority research.

**Thank you for making this very important point. We have drastically increased the period of time covered in this manuscript. We expected, as you said, that vaccination and improved treatment options for COVID-19 would mean that mortality from concurrent TB/COVID-19 was decreased, or even largely the same as mortality among TB-alone patients, when we examined the entire period since the start of the pandemic. Instead, we were surprised to find that extending the timeline only strengthened our findings. We agree that this makes this research much more impactful than it was before. Now, instead of saying that concurrent TB/COVID-19 was a dangerous combination in the early days of the pandemic, we can say that it appears to be a dangerous combination over the entire period from March 2020 to June 2022. When looking at Figure 1, we can see that deaths among patients with concurrent TB/COVID-19 continue to occur frequently. We have added the following line to discuss these points:**

Line 340: Even after doing sensitivity checks on how we defined “concurrent” TB/COVID-19 (as diagnoses within 120, 90, 60, or 30 days) and controlling for patient age and sex, death rates were significantly higher among patients with concurrent TB/COVID-19 than among patients with TB-alone. It is surprising that the percentage of patients with concurrent TB/COVID-19 who died was so high, given that the period covered included not only the “lockdown” period in NYC, but also 18 months in 2021 and 2022 in which vaccination and access to anti-viral and other treatments became increasingly widespread.

6. PLOS authors have the option to publish the peer review history of their article ([what does this mean?](https://journals.plos.org/globalpublichealth/s/editorial-and-peer-review-process#loc-peer-review-history)). If published, this will include your full peer review and any attached files.


**Do you want your identity to be public for this peer review?** If you choose “no”, your identity will remain anonymous but your review may still be made public.


For information about this choice, including consent withdrawal, please see our [Privacy Policy](https://www.plos.org/privacy-policy).

Reviewer #1: No

Reviewer #2: No

[NOTE: If reviewer comments were submitted as an attachment file, they will be attached to this email and accessible via the submission site. Please log into your account, locate the manuscript record, and check for the action link "View Attachments". If this link does not appear, there are no attachment files.]

While revising your submission, please upload your figure files to the Preflight Analysis and Conversion Engine (PACE) digital diagnostic tool, https://pacev2.apexcovantage.com/. PACE helps ensure that figures meet PLOS requirements. To use PACE, you must first register as a user. Registration is free. Then, login and navigate to the UPLOAD tab, where you will find detailed instructions on how to use the tool. If you encounter any issues or have any questions when using PACE, please email PLOS at figures@plos.org. Please note that Supporting Information files do not need this step.

*In compliance with data protection regulations, you may request that we remove your personal registration details at any time.* [*(Remove my information/details)*](https://www.editorialmanager.com/pgph/login.asp?a=r)*. Please contact the publication office if you have any questions.*

1.	Krishnan JK, Rajan M, Baer BR, Hoffman KL, Alshak MN, Aronson KI, et al. Assessing mortality differences across acute respiratory failure management strategies in Covid-19. J Crit Care. 2022;70:154045.

2.	Perkins GD, Ji C, Connolly BA, Couper K, Lall R, Baillie JK, et al. Effect of Noninvasive Respiratory Strategies on Intubation or Mortality Among Patients With Acute Hypoxemic Respiratory Failure and COVID-19: The RECOVERY-RS Randomized Clinical Trial. JAMA. 2022;327(6):546-58.
